# End-stage heart failure and heart transplant in cardiac sarcoidosis: a case series

**DOI:** 10.1093/ehjcr/ytae635

**Published:** 2024-12-03

**Authors:** Maria Francesca Scuppa, Antonella Accietto, Anna Corsini, Maddalena Graziosi, Elena Biagini, Chiara Baldovini, Mario Sabatino, Luciano Potena

**Affiliations:** Cardiology Unit, IRCCS Azienda Ospedaliero-Universitaria di Bologna, Policlinico Sant’Orsola Malpighi, Via Massarenti 9, 40138 Bologna, Italy; Department of Medical and Surgical Sciences, University of Bologna, Via Massarenti 9, 40138 Bologna, Italy; Cardiology Unit, IRCCS Azienda Ospedaliero-Universitaria di Bologna, Policlinico Sant’Orsola Malpighi, Via Massarenti 9, 40138 Bologna, Italy; Department of Medical and Surgical Sciences, University of Bologna, Via Massarenti 9, 40138 Bologna, Italy; Cardiology Unit, IRCCS Azienda Ospedaliero-Universitaria di Bologna, Policlinico Sant’Orsola Malpighi, Via Massarenti 9, 40138 Bologna, Italy; Cardiology Unit, IRCCS Azienda Ospedaliero-Universitaria di Bologna, Policlinico Sant’Orsola Malpighi, Via Massarenti 9, 40138 Bologna, Italy; Cardiology Unit, IRCCS Azienda Ospedaliero-Universitaria di Bologna, Policlinico Sant’Orsola Malpighi, Via Massarenti 9, 40138 Bologna, Italy; Pathology Unit, IRCCS Azienda Ospedaliero-Universitaria di Bologna, Via Massarenti 9, 40138 Bologna, Italy; Heart Failure and Transplant Unit, IRCCS Azienda Ospedaliero-Universitaria di Bologna, Via Massarenti 9, 40138 Bologna, Italy; Heart Failure and Transplant Unit, IRCCS Azienda Ospedaliero-Universitaria di Bologna, Via Massarenti 9, 40138 Bologna, Italy

**Keywords:** Cardiac sarcoidosis, Heart transplant, Heart failure, Inflammatory cardiomyopathy, Case series

## Abstract

**Background:**

Diagnosing cardiac sarcoidosis (CS) is challenging. Immunosuppressive therapies are less effective in end-stage disease, and often heart transplant (HT) is the only available option. We present a series of advanced CS cases, requiring HT, along with a review of the literature evidence in this field.

**Case summary:**

Case 1: a 49-year-old man initially suspected of having arrhythmogenic cardiomyopathy (ACM) presented with heart failure (HF) and recurrent ventricular arrhythmias. The rapid clinical deterioration raised suspicion of an inflammatory aetiology, which was confirmed through endomyocardial biopsy, diagnosing CS. Despite immunosuppressive therapy, HT was required. Case 2: a 36-year-old woman presented with high-grade atrioventricular block and dilated cardiomyopathy (DCM), initially diagnosed as idiopathic. Due to worsening HF, she required HT. The pathological examination of the explanted heart revealed CS. Chronic subclinical antibody-mediated rejection was observed after HT. Case 3: a 44-year-old man presented with syncope and imaging suggesting ACM. He was referred for HT due to high ventricular arrhythmic burden. Cardiac sarcoidosis diagnosis was suspected due to pulmonary involvement and then confirmed on post-explant pathological exam. Post-HT pulmonary and cutaneous sarcoidosis reactivation were observed. Case 4: a 43-year-old man was diagnosed with pulmonary sarcoidosis after lung biopsy. Progression towards DCM was observed despite immunosuppressive therapy. Post-HT was characterized by multiple episodes of graft rejection.

**Discussion:**

This case series provides insights into mid- and long-term outcomes after HT for CS, highlighting the need for careful management of immunosuppression in these patients, balancing the adverse effects of chronic immunosuppression with the prevention of rejection and sarcoidosis recurrence.

**Learning points**

- Cardiac sarcoidosis is difficult to diagnose, especially in advanced cases. Suspicion of inflammatory cardiomyopathy should arise in case of rapid progression or unexplained arrhythmic manifestations or heart failure. Definite diagnosis is achieved only with endomyocardial biopsy, although false negatives may occur due to the patchy distribution of myocardial inflammation. A highly probable diagnosis of cardiac sarcoidosis should be made by combining clinical, imaging, and pathological criteria.
- Choosing the right therapeutic option is also challenging. In advanced cases, immunosuppressive therapy is not always successful in reducing the arrhythmic burden and improving cardiac contractility; therefore, heart transplant often remains the only strategy.
- Post-transplant risk of rejection and systemic sarcoidosis relapse, as well as the risks of long-term immunosuppression, are to be taken into consideration. Post-transplant complications observed in our cohort were graft rejection and reactivation of systemic sarcoidosis, notably when immunosuppressive treatment was temporarily lowered.

## Introduction

Sarcoidosis is an inflammatory disease of unknown origin, characterized by the presence of non-caseating epithelioid cell granulomas, which can affect any organ. Intrathoracic involvement occurs in over 90% of cases, with the lungs being the most affected organ. Cardiac involvement may present in multisystemic sarcoidosis or as the initial and sole manifestation of the disease, in ∼25% of the cases.^1^ Cardiac sarcoidosis (CS) prevalence varies depending on age, ethnicity, and the diagnostic methods used. While CS is clinically manifest in only 5% of patients with systemic sarcoidosis, with cardiac imaging techniques, such as cardiac magnetic resonance (CMR) imaging and 18-fluoro-2-deoxyglucose positron emission tomography (^18^FDG-PET), cardiac involvement rises to 20%.^1,2^

Cardiac sarcoidosis is an infiltrative cardiomyopathy, and its clinical manifestations depend on the location and extent of granulomas. Due to its diverse and non-specific presentations, CS can be challenging to diagnose. ‘Red flags’ for CS include high-grade atrioventricular block (AVB) in younger patients, unexplained ventricular arrhythmias (VAs), reduced left ventricular (LV) ejection fraction (LVEF), regional wall aneurysms, or basal septal thinning observed on echocardiography.^2^

Over time, diagnostic criteria for CS have been issued by multiple societies (*Table 1*).^3–6^ Although a definite diagnosis is established with histological evidence of non-caseating granulomas in the heart, combining clinical, imaging, and pathological data can achieve a highly probable diagnosis.

**Table 1:** Diagnostic criteria for cardiac sarcoidosis, elaborated by the Heart Rhythm Society, the World Association of Sarcoidosis and Other Granulomatous Disorders, and the Japanese Circulation Society

HRS criteria (2014)​^4^	WASOG criteria (2014)​^5^	JCS criteria (2016)​^6^
Histological diagnosis:​		
EMB or surgical specimens demonstrate non-caseating granulomas​		
Clinical diagnosis:​	Clinical diagnosis:​	Clinical diagnosis:​
Histological diagnosis of extracardiac sarcoidosis​	Granulomatous inflammation demonstrated in another organ	Histological diagnosis of extracardiac sarcoidosis​
AND ​		
		AND ​
	AND​	
One of the following:​	One of the following: ​	≥2 major criteria OR 1 major and ≥2 minor criteria​
		Major criteria: ​
Immunosuppressant-responsive cardiomyopathy or heart block​ Unexplained reduced LVEF < 40%​ Unexplained sustained VT or high-degree AVB​ Patchy ^18^FDG uptake on a dedicated cardiac PET in a pattern consistent with CS​ LGE on CMR in a pattern consistent with CS​ Positive 67 Ga uptake in a pattern consistent with CS​	Treatment-responsive cardiomyopathy and AVB ​ Reduced LVEF in the absence of other risk factors ​ Spontaneous or inducible sustained VT with no risk factors ​ High-degree AVB ​ Patchy uptake on a dedicated cardiac PET LGE on CMR ​ Positive 67 Ga uptake ​ Defect on perfusion scintigraphy or SPECT scan ​ T2 prolongation on CMR ​	High-degree AVB or fatal VT/VF ​ Basal thinning of the ventricular septum or abnormal ventricular wall anatomy ​ LV contractile dysfunction ​ 67 Ga or ^18^FDG-PET reveals abnormally high tracer uptake in the heart ​ CMR reveals LGE of the myocardium ​
		Minor criteria: ​
		Abnormal ECG findings (non-sustained VT, premature ventricular complexes, bundle-branch block, axis deviation, and abnormal Q waves) ​ Perfusion defects on SPECT ​ Monocyte infiltration and moderate fibrosis on EMB
AND alternative causes have been reasonably excluded​	​AND alternative causes have been reasonably excluded​	AND exclusion of coronary artery disease and other cardiomyopathies​

Adapted from Sharma *et al*.^3^

AVB, atrioventricular block; CS, cardiac sarcoidosis; CMR, cardiac magnetic resonance; ECG, electrocardiography; EMB, endomyocardial biopsy; ^18^FDG, 18-fludeoxyglucose; HRS, Heart Rhythm Society; JCS, Japanese Circulation Society; LGE, late gadolinium enhancement; LVEF, left ventricular ejection fraction; PET, positron emission tomography; SPECT, single photon emission computed tomography; VF, ventricular fibrillation; VT, ventricular tachycardia; WASOG, World Association of Sarcoidosis and Other Granulomatous Disorders.

Despite limited evidence, immunosuppression, with corticosteroids as first-line treatment, is the cornerstone of CS therapy.^1,2^ However, the response to treatment may not be as effective in advanced stages.^7,8^

Cardiac involvement conveys a worse prognosis; in a retrospective cohort of 512 CS patients, the long-term incidence of adverse events was high: 10-year incidence of all-cause death was 18%, hospitalization for heart failure (HF) 21%, and VAs 31.9%.^9^ In another retrospective cohort, 5% underwent heart transplant (HT) over 20 years; cardiac involvement was associated with a more advanced presentation and a worse outcome.^10^ However, the rate of HT varies in different cohorts.^1^

We report four cases of patients referred to our advanced HF unit, where the diagnosis was delayed because of fast progression, late onset of symptoms, or misdiagnosis, and for which HT represented the only viable therapeutic option. We aim to share our experience in HT management and post-transplant outcomes in CS, while providing a review of the literature already available (*Table 2*).

**Table 2:** Brief review of the literature available on heart transplantation in cardiac sarcoidosis patients

Author	*n* of patients (CS/total)	Provenience	Time interval	CS diagnosis	Characteristics (CS patients compared with non-CS patients)	Prognosis (survival, redo HT) (CS patients compared with non-CS patients)	Rejection (CS patients compared with non-CS patients)	Recurrence	IS regimen
Akashi *et al.*^11^	14/825	Monocentric	1997–2010	6/14 pre-HT diagnosis: 2/14 EMB, 4/14 extracardiac involvement 8/14 explanted heart	CS patients: more likely females, Black	Survival at 1 and 5 years: 1 year: 78.5% vs. 87.2%	CS: 4/14 ACR	2/14 graft recurrence	CNI + MMF + Prednisone
		USA							
		Columbia University Medical Center							
					7/14 extracardiac sarcoidosis (100% pulmonary)	5 years: 58.9% vs. 76.2% (*P* = 0.09)			
Bobbio *et al.*^12^	11/66 (matched 1:5)	Monocentric	1993–2018	4/11 EMB	4/11 extracardiac sarcoidosis	Survival at 1 and 5 years: No difference	CS: 1/11 ACR	2/11 graft recurrence	CNI + MMF + Prednisone
		Sweden		7/11 explanted heart					
							No difference		
		Sahlgrenska University Hospital							
Chang *et al.*^13^	5/411	Monocentric	1987–2011	1/5 EMB	5/5 isolated cardiac sarcoidosis	Survival at 1 and 5 years: No deaths in the CS group, no data on non-CS population	NA	No recurrence	Induction regimen: ATG maintenance: CYA + AZA + Prednisone
		Taiwan		4/5 explanted heart					
		National Taiwan University Hospital							
Crawford *et al.*^14^	67/18347	Multicentric	2006–15	NA	CS patients: more likely females, Black	Survival at 1 and 5 years:	NA	NA	NA
		USA							
						1 year:			
		UNOS							
					18/67 bridged with LVAD, 1/67 bridged with TAH, 1/67 bridged with BiVAD	91% vs. 90% (*P* = 0.88)			
						5 year: 83% vs. 77% (*P* = 0.46)			
McGoldrick *et al.*^15^	289/41447	Multicentric	1999 to March 2020	NA	CS patients: more likely females, Black, younger t-MCS less frequent, urgent HT more frequent (1A-1B UNOS status)	Survival at 10 years: 73.4% vs. 59.5% (*P* = 0.002)	No difference	NA	NA
		USA							
		UNOS							
						Retransplantation: No difference			
Perkel *et al.*^16^	19/1069	Single centre	1991–2010	7/19 EMB	8/19 extracardiac sarcoidosis	Survival at 5 years: 79% vs. 83% (*P* = NS)	No difference	No recurrence on graft	CNI + MMF/AZA + Prednisone
		USA	Last FUP July 2011	12/19 explanted heart					
		Cedars Sinai							
							No difference in CAV	3/19 extracardiac recurrence (2 lung, 1 liver)	
Rosenthal *et al.*^17^	12/24 (matched)	Single centre	1995–2016	NA	CS patients: more likely males	Post-HT survival: No deaths in the CS group	CS: 2/12 ACR (vs. 19/21 non-CS, *P* < 0.01)	No recurrence	Induction regime: ATG maintenance:
		USA Washington Medical Centre							
									TAC + MMF + Prednisone
					LVAD less frequent				
					5/12 isolated CS				
							Less rejection in CS (17% vs. 68%, *P* = 0.006)		
Theofilogiannakos *et al.*^18^	12/901	Single centre	1990–2012	4/12 extracardiac (1 cutaneous, 3 lung)	CS patients: younger tMCS less frequent	Survival at 1 and 5 years in CS: 92% and 83%. No difference with non-CS	CS:	No recurrence	CNI + AZA/MMF + Prednisone
							3/12 ACR		
		UK Papworth Hospital	Last FUP: 1/06/2014						
							3/12 CAV		
					Cardiac manifestations: 12/12 DCM	In CS group: No retransplantation			
						No malignancy			
					2/12 VA				
					1/12 high-grade AVB				
Velikanova *et al.*^19^	15/502	Multicentric Finland	Late 1980s to 2015	7/15 explanted heart	15/15 advanced HF	Survival at 1 and 5 years: No difference	CS: 4/15 ACR	No recurrence	NA
					1/15 urgent HT				
					1/15 VA				
Zaidi *et al.*^20^	65/38230	Multicentric	October 1987 to September 2005	NA	CS patients: more likely males, Black	Survival at 1 and 5 years: 87.7% vs. 84.5% (*P* = 0.03)	No difference	NA	NA
		USA							
		UNOS							
						In CS: 2/65 retransplantation			

ACR, acute cellular rejection; ATG, antithymocyte globulin; AVB, atrioventricular blocks; AZA, azathioprine; Bi-VAD, biventricular assist device; CAV, coronary allograft vasculopathy; CYA, cyclosporine; CNI, calcineurin inhibitors; CS, cardiac sarcoidosis; DCM, dilated cardiomyopathy; EMB, endomyocardial biopsy; FU, follow up; HF, heart failure; HT, heart transplant; IS, Immunosuppressive; LVAD, left ventricle assist device; MMF, mycophenolate mofetil; NA, not available; TAC, tacrolimus; TAH, total artificial heart; t-MCS, temporary mechanical support; UNOS, United Network for Organ Sharing; VA, ventricular arrhythmia.

## Summary figure

**Figure:**
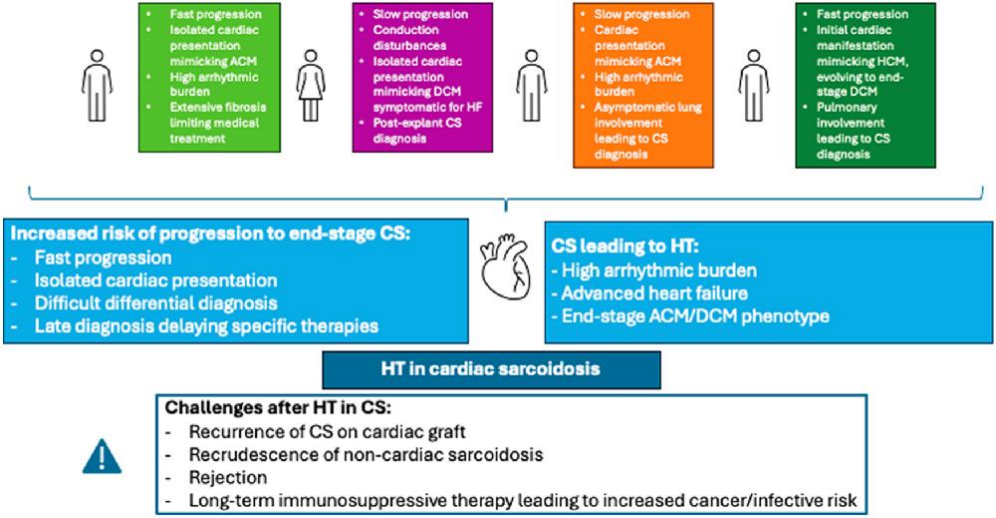


## Patient 1

**Figure 1:**
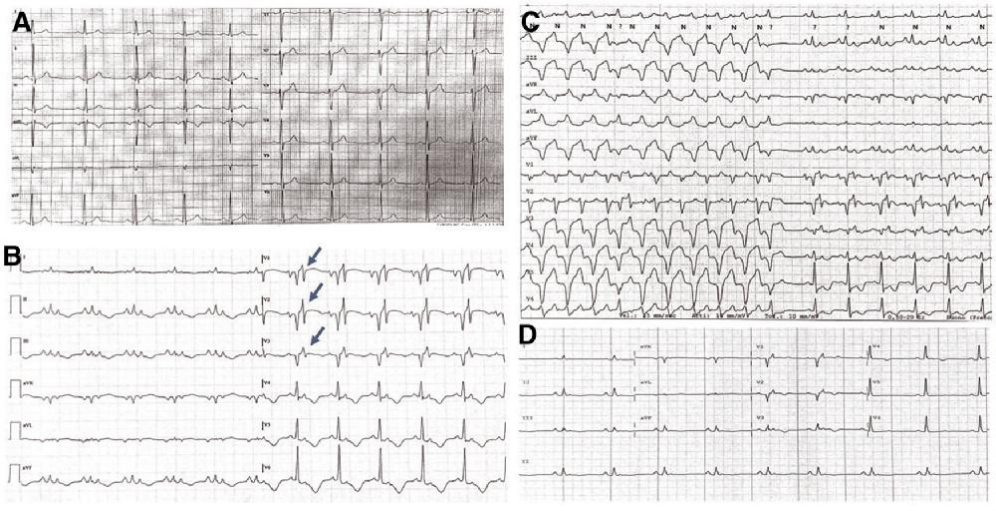
(Patient 1) Electrocardiogram evolution. (*A*) Electrocardiogram 2 years before: sinus rhythm, normal atrioventricular and interventricular conduction, normal repolarization. (*B*) Electrocardiogram at intensive care unit admission: left atrial enlargement, QRS potential reduction, extreme QRS prolongation and fragmentation, prominent epsilon wave in right precordial leads (arrows), and negative infero-lateral T waves. (*C*) Beats of ventricular tachycardia probably originating from the right ventricular apex and terminating in sinus rhythm. (*D*) Electrocardiogram after 2 months of immunosuppressive therapy: partial regression of electrocardiogram changes: persistence of voltage reduction and epsilon waves in right precordial leads (adapted from Chietera and Biffi^21^).

A 49-year-old man with no significant medical history was admitted in a community hospital due to palpitations and worsening dyspnoea (New York Heart Association – NYHA III). Electrocardiogram (ECG) showed a wide and fragmented QRS complex with prominent epsilon waves (*Figure 1B*); echocardiography showed an LVEF of 45%, and mild right ventricular (RV) dysfunction. Coronary angiography ruled out a coronary artery disease. Cardiac magnetic resonance suggested biventricular arrhythmogenic cardiomyopathy (ACM). In the following weeks, the patient was readmitted due to rapidly worsening HF symptoms. The ECG revealed sustained ventricular tachycardia (VT) treated with DC shock. Recurrent VT occurred, with only partial response to amiodarone, lidocaine, and procainamide (*Figure 1C*). Due to electric and haemodynamic instability, the patient was transferred to our intensive care unit (ICU), where an intra-aortic balloon pump (IABP) was implanted.

**Figure 2:**
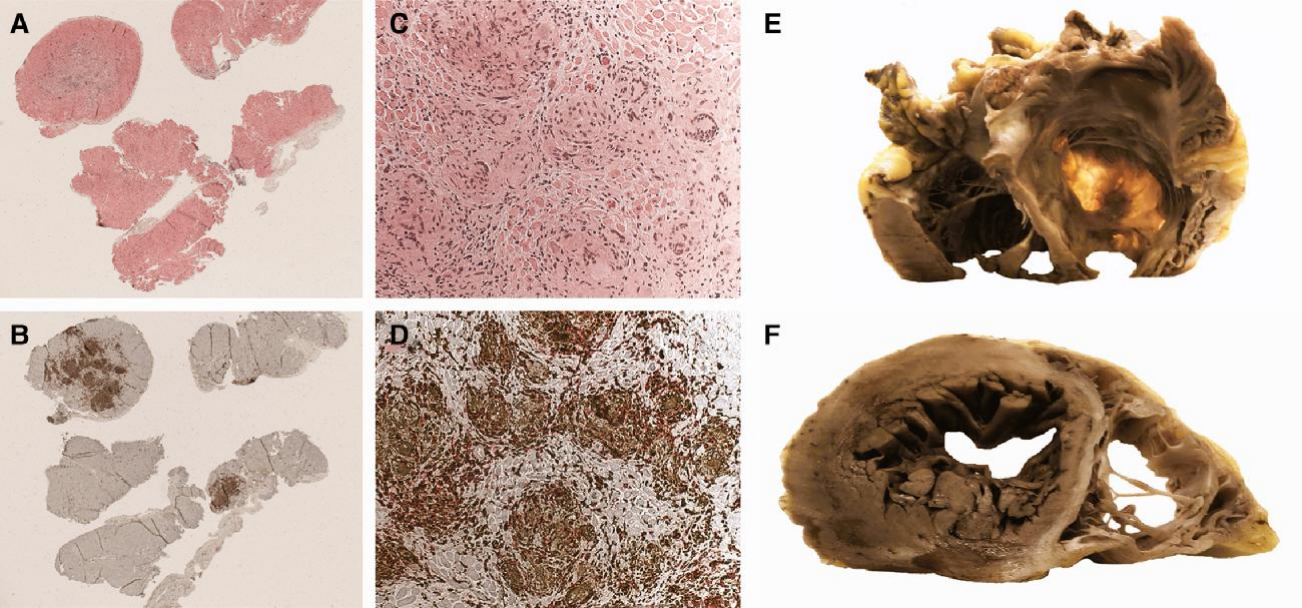
(Patient 1) Endomyocardial biopsy and explanted heart. (*A* and *B*) Inflammatory process with multifocal distribution (*A*: haematoxylin-eosin 25×; *B*: double CD68/CD3 immunostaining, CD68+ macrophages in brown, CD3 T lymphocytes in red 25×). (*C* and *D*) Macrophagic non-necrotizing granulomas and giant cells, associated with T lymphocytes (*C*: haematoxylin-eosin 200×; *D*: double CD68/CD3 immunostaining 200×). (*E*) Aneurysmal dilatation of the right ventricular outflow tract, shown by transillumination. (*F*) On the short-axis section, both ventricles are dilated; the free walls of the right ventricle and the septum are thinned and display large areas of fibrous replacement.

**Figure 3:**
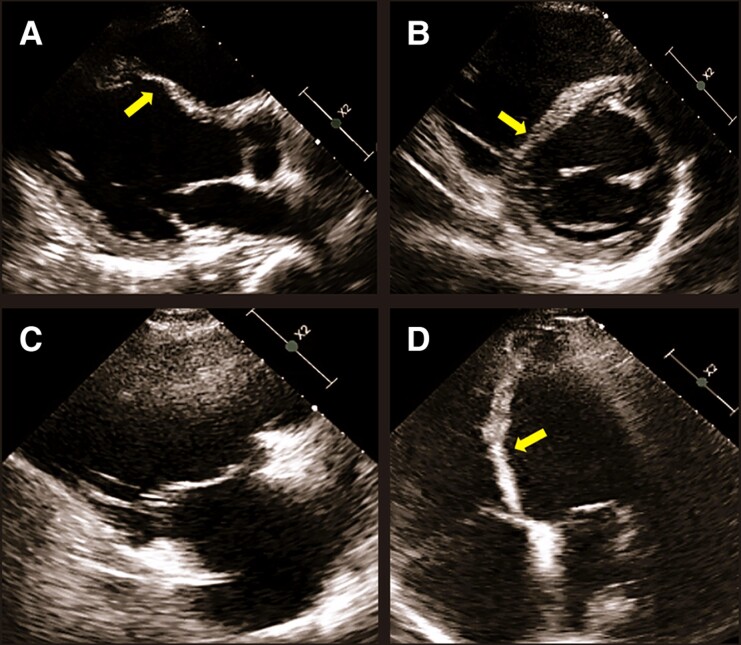
(Patient 1) Echocardiogram. Transthoracic echocardiography showing a moderately dilated and severely hypokinetic left ventricle (index end-diastolic volume 92 mL/m^2^, ejection fraction 27%) with thinned and dyskinetic mid-basal interventricular septum (*A, B*, and *D*, arrow). The right ventricle was severely dilated and akinetic, notably in the inferior wall (end-diastolic area 36 cm^2^ and fractional area change 13%) (*C*).

**Figure 4:**
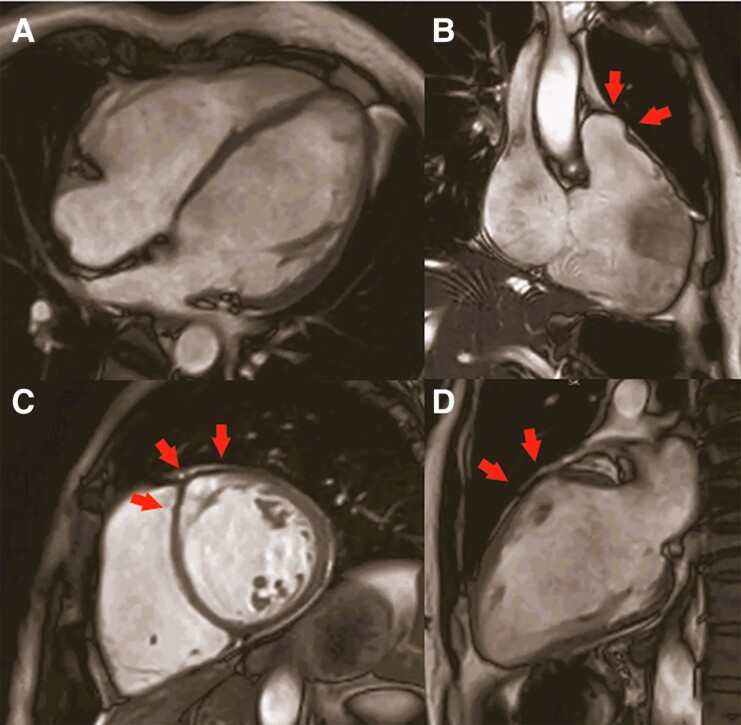
(Patient 1) Cardiac magnetic resonance frame images from cine steady-state free precession. Dilated and hypokinetic left ventricle (index end-diastolic volume 135 mL/m^2^ and ejection fraction 21%; *A*) and severe dilatation and hypokinesia of the right ventricle (index end-diastolic volume 160 mL/m^2^, ejection fraction 24%) with a systolic bulging of the anterior wall (*B*, arrows). (*C* and *D*) Akinetic and thinned anterior left ventricular interventricular septum and anterior wall at basal segments (wall thickness 2.8 mm) with aneurysmatic dilatation (*C* and *D*, red arrows), a specific clue for cardiac sarcoidosis.

**Figure 5:**
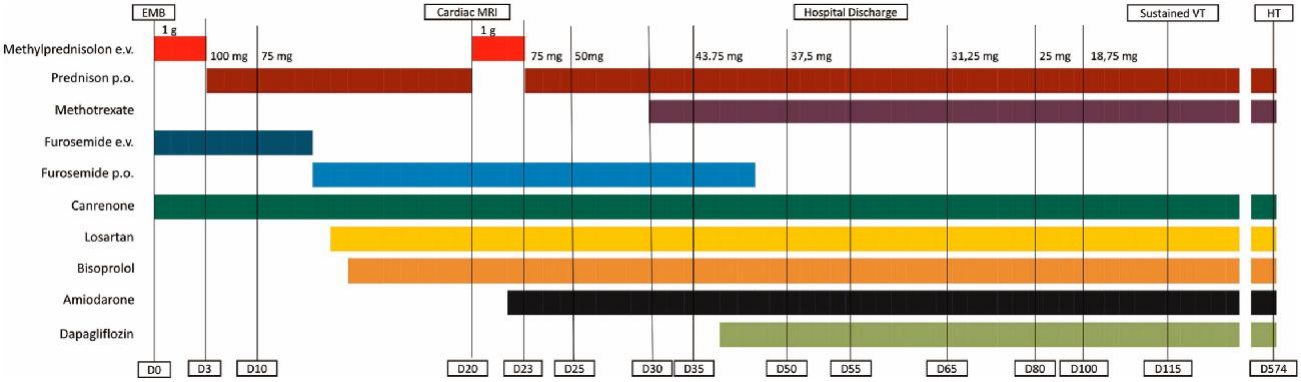
(Patient 1) Therapy timeline. Evolution of therapy chart from Day 0 to transplant date (Day 574). Of note, immunosuppressive regimen consisted of initially pulsed intravenous high dose corticosteroid; then, from Day 3, oral steroid therapy was started and gradually tapered. After cardiac magnetic resonance was performed, the immunosuppressive regime was implemented by adding a steroid-sparing drug (methotrexate). Meanwhile, heart failure-specific therapy was titrated, and antiarrhythmic therapy (amiodarone) was introduced. Diuretic therapy was slowly reduced, according to improved fluid status. CMR, cardiac magnetic resonance; EMB, endomyocardial biopsy; D, day; HT, heart transplant; VT, ventricular tachycardia.

Given the rapid clinical deterioration, we suspected an inflammatory trigger and we performed an endomyocardial biopsy (EMB). It revealed a multifocal inflammatory process (*Figure 2A* and *B*) with non-necrotizing granulomas containing macrophages and multinucleated giant cells and mild fibrosis, consistent with CS (*Figure 2C* and *D*). Different imaging techniques showed severe biventricular dysfunction and identified a mid-basal interventricular focal aneurysm, a feature suggestive of CS (*Figures 3* and *4*). Additionally, ^18^FDG-PET detected isolated cardiac uptake, confirming the diagnosis of CS, and excluded extracardiac involvement. Corticosteroid treatment was initiated (*Figure 5*), with progressive clinical improvement.

After 1 month, CMR showed persistent biventricular inflammation, with no improvement in biventricular function and methotrexate was added. A follow-up EMB showed inflammation resolution with extensive fibrosis.

Given the lack of improvement in LV function despite immunosuppressive therapy, the patient was placed on HT waiting list.

Three months later, the patient received an HT. The explanted heart was dilated, with severe thinning and fibrous replacement of the RV free wall and with aneurysmatic dilatation of the infundibulum. The septum was significantly thinned with extensive fibrosis (*Figure 2E* and *F*). Histological examination showed large areas of fibrous replacement, involving the RV and the basal septum and scattered inflammatory infiltrates devoid of well-formed granulomas within the fibrous tissue. We suspect that the intensive immunosuppressive treatment reduced inflammation, leaving extensive scar.

After HT, the patient received antithymocyte globulin followed by standard immunosuppressive therapy with tacrolimus, mycophenolate mofetil (MMF), and prednisone, without rejection or sarcoid relapse. Patient is alive and clinically stable 2 years after HT.

## Patient 2

A 36-year-old woman with no previous medical history presented to the emergency department for palpitations. Previously unknown atrial fibrillation was documented. At that time, echocardiography was normal.

Two years later, the patient underwent pacemaker implantation due to high-grade AVB. A follow-up ultrasound revealed LV dilation and reduced LVEF. No coronary artery disease was found on coronary angiography. Therefore, idiopathic dilated cardiomyopathy (DCM) was diagnosed.

Despite optimal medical treatment, she developed refractory HF and was therefore listed for HT. Post-explant pathological exam was suggestive for CS, with extensive areas of active inflammation and non-caseating granulomas alternating with areas of myocardial necrosis and transmural fibrosis. After HT, an immunosuppressive regime with cyclosporine, MMF, and prednisone was started. Three years after HT, Class I donor-specific antibodies (DSAs) were detected, without evidence of graft dysfunction or rejection on EMB. Both extracardiac and graft sarcoidosis were excluded. Fourteen years after HT, she was hospitalized for HF with severe systolic dysfunction (LVEF 35%) and newly detected Class II DSA. Endomyocardial biopsy ruled out acute rejection or sarcoid recurrence, but chronic subclinical antibody-mediated rejection (AMR) was diagnosed. Intravenous steroids, high-dose diuretics, and inotropes were started. However, her condition continued to deteriorate, she developed sepsis, and she ultimately died in the ICU from mixed (cardiogenic and septic) shock.

## Patient 3

A 44-year-old man with a long-standing history of ventricular ectopies was hospitalized for syncope. Electrocardiogram showed a first-grade AVB with right bundle branch block, and cardiac ultrasound showed moderate isolated RV systolic dysfunction. Coronary angiography revealed no lesions. Cardiac magnetic resonance was suggestive of ACM. The patient underwent ICD implantation, but despite antiarrhythmic therapy and endocardial ablation, he experienced multiple appropriate DC shocks for sustained VT. Due to the arrhythmic burden, he was referred for HT. Screening chest CT showed multiple perilymphatic pulmonary micronodules and several mediastinal lymph nodes with rim-like contrast enhancement. 18-Fluoro-2-deoxyglucose positron emission tomography showed lungs and lymph node uptake. Lymphnodal biopsy confirmed active sarcoidosis. Steroidal therapy was started. Right ventricular EMB showed diffuse fibrosis without signs of inflammation or non-caseating granulomas.

After HT, he received immunosuppressive therapy with prednisone, MMF, and cyclosporine. Pathology of the explanted heart confirmed CS. One month later, MMF was discontinued due to leukopenia. Three weeks afterwards, the patient reported worsening fatigue and development of multiple painful subcutaneous nodules, leading to a diagnosis of cutaneous sarcoidosis. Additionally, pneumological evaluation, chest CT and ^18^FDG-PET revealed pulmonary sarcoidosis reactivation. Corticosteroid dosage was increased, resulting in the progressive resolution of skin lesions. After 6 months, ^18^FDG-PET revealed no uptake and chest CT showed partial regression of the micronodules. Immunosuppressive therapy with prednisone (7.5 mg daily) and cyclosporine was continued, and the patient has remained clinically stable with no significant events 4 years after HT.

## Patient 4

A 43-year-old man underwent cardiac evaluation following a transient ischaemic attack. His family and past medical history were unremarkable. Cardiac ultrasound revealed LV hypertrophy and pulmonary hypertension. A chest CT showed multiple bilateral micronodules and a lung biopsy was diagnostic for sarcoidosis, leading to the initiation of steroid therapy. Over the next 2 years, the patient experienced a progressive decline, culminating in an end-stage DCM (LVEF 25%) and intraventricular conduction delay, which required cardiac resynchronization therapy with defibrillator implantation. Active pulmonary and CS were excluded with ^18^FDG-PET and chest CT. Despite optimal HF medical treatment, LV dysfunction persisted.

Due to rapidly worsening HF, requiring IABP and inotropic support, the patient underwent an urgent HT. Post-transplant, he received a standard immunosuppressive regimen consisting of tacrolimus, MMF, and prednisone.

Two months after HT, MMF was discontinued due to leukopenia. Subsequently, the patient experienced multiple episodes of graft rejection with negative DSA and no evidence of CA recurrence. These episodes required multiple lines of immunosuppressive treatment (intravenous corticosteroids, immunoglobulins, and extracorporeal photopheresis). Following cycles of extracorporeal photopheresis, the EMB confirmed the resolution of rejection. The patient is alive 4 years after HT, with no evidence of sarcoidosis recurrence or infectious complications.

## Discussion

Diagnosing CS can be particularly challenging, especially in advanced or isolated cases. Cardiac sarcoidosis can present with diverse clinical manifestations, depending on the disease's localization, extent, and activity (e.g. VA in Patients 1 and 3, high-grade AVB in Patient 2, and HF in Patient 4). Other cardiomyopathies, such as ACM in Patients 1 and 3 and DCM in Patients 2 and 4, can mimic CS, complicating the diagnosis.

Several diagnostic algorithms are available (*Table 1*) to facilitate the diagnostic process. While definite diagnosis is achieved only with EMB, it has limitations due to the patchy distribution of the myocardial involvement, which can lead to false negatives. A highly probable CS diagnosis should be made by combining clinical, imaging, and pathological criteria.^4–6^

**Figure 6:**
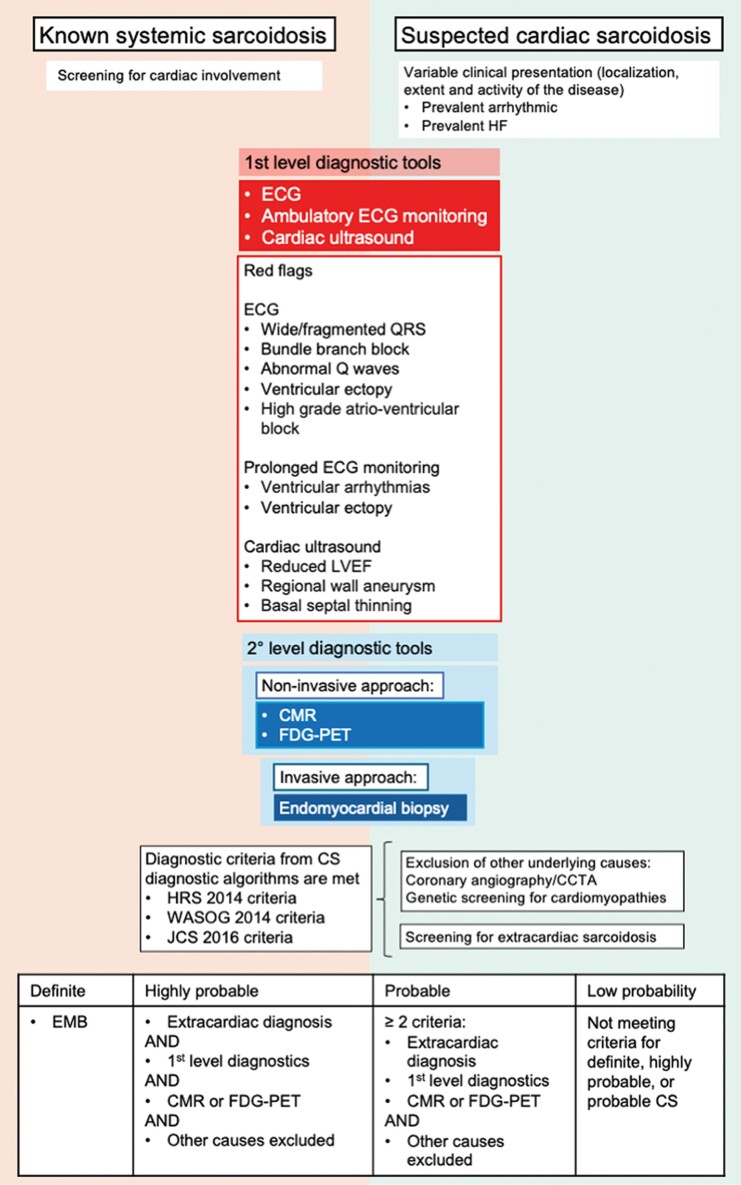
Cardiac sarcoidosis can be investigated in the context of known systemic sarcoidosis or in the setting of a new cardiac condition. First-level diagnostic tools such as electrocardiogram and cardiac ultrasound can be suggestive for cardiac sarcoidosis, thus prompting investigation with more advanced instruments: non-invasive multimodality imaging and invasive pathological exam. In dubious cases, diagnostic criteria can help to reach a definite diagnosis. CCTA, cardiac computed tomography angiography; CMR, cardiac magnetic resonance; CS, cardiac sarcoidosis; ECG, electrocardiography; EMB, endomyocardial biopsy; FDG-PET, fluorodeoxyglucose positron emission tomography; HF, heart failure; HRS, Heart Rhythm Society; JCS, Japanese Circulation Society; WASOG, World Association of Sarcoidosis and Other Granulomatous Disorders.

First-line diagnostic tools, like ECG and echocardiography, although limited in sensitivity, can raise suspicion of CS (see ‘red flags’; *Figure 6*). When CS is suspected, second-line diagnostic tools are required. Cardiac magnetic resonance detects late gadolinium enhancement (LGE), indicating acute inflammation (oedema/necrosis) or chronic evolution (fibrosis/scar). However, no specific or diagnostic LGE pattern exists for CS. Cardiac magnetic resonance has a high negative predictive value, with false negatives mostly occurring in early disease stages. Differentiating between ACM and CS can be difficult, but rapidly evolving ECG features, the presence of basal septal aneurysm, and massive RV LGE at CMR should orient towards the CS.

18-Fluoro-2-deoxyglucose positron emission tomography detects metabolically active areas compatible with inflammation, with multifocal FDG uptake being a hallmark for CS. Therefore, ^18^FDG-PET yields a high positive predictive value, with false negatives mainly seen in advanced, ‘burned out’ cases. Cardiac magnetic resonance and ^18^FDG-PET can be used synergistically, especially in uncertain cases.^2^

When a definite diagnosis is not reached despite a complete multimodality evaluation, genetic testing can be helpful in identifying specific genetic mutations associated with DCM or ACM (e.g. desmoplakin cardiomyopathy).

In known systemic sarcoidosis, early identification of cardiac involvement is crucial, due to its impact on prognosis. Regular cardiovascular evaluations, including first-line exams (ECG, Holter monitoring, and echocardiogram), should be performed to detect subclinical or early cardiac involvement. Second-line tests should be considered if CS is suspected (*Figure 6*).^2^

The four cases illustrate how delayed diagnosis, due to inadequate cardiac screening in patients with systemic sarcoidosis (Patient 4), failure to recognize red flags (Patient 2), or misdiagnosis with other cardiomyopathies (Patients 1 and 3), can adversely affect outcomes. Even with appropriate immunosuppressive therapy, late treatment may not reduce arrhythmic burden or improve cardiac function, leaving HT as the only viable long-term option.

Immunosuppressive therapy is the cornerstone of active CS treatment, with corticosteroids as the first-line option. Antimetabolites can be added in cases of inadequate response, relapse after tapering, to reduce the effective steroid dose, or as a starting therapy in severe CS manifestations. Anti-TNF/anti-CD20 monoclonal antibodies can be considered as a third-line steroid-sparing agents.^3^

In patients with HF, guideline-directed medical and device therapy should be administered. Long-term mechanical circulatory support (lt-MCS) and HT are options for end-stage HF. However, advanced HF therapies in CS patients require careful consideration of specific factors. Extensive extracardiac involvement may be a barrier to HT or impact post-HT outcomes. Additionally, the potential toxicity of long-term immunosuppression, before and after HT in patients with CS, should be carefully evaluated. For candidates to lt-MCS certain conditions must be closely assessed. These include the presence of a restrictive phenotype, a high burden of VAs or extensive RV involvement, as they can influence implantation feasibility, device choice, and overall outcomes.^2^

Survival, rejection, and recurrence rates after HT in CS patients are encouraging, although the existing literature predominantly comprises case reports and small case series (*Table 2*). Overall, post-HT survival and rejection rates in CS patients are comparable with non-CS patients, and the recurrence rate of CS in the transplanted heart is generally low.

Post-transplant care in CS patients, particularly regarding immunosuppression and recurrence monitoring, lacks clear guidelines. In our cohort, two patients experienced graft rejection: one case of chronic subclinical AMR and one case of ACR with negative DSA in the context of reduced immunosuppression. Notably, a systemic relapse of sarcoidosis was observed when immunosuppressive treatment was temporarily lowered.

These findings highlight the importance of carefully managing immunosuppression in CS patients after HT, balancing the adverse effects of chronic immunosuppression with the need to prevent rejection and sarcoidosis recurrence. Further research is necessary to establish clear guidelines for optimal post-transplant care in this population.

## Data Availability

All data are incorporated into the article.

## Lead author biography

Maria Francesca Scuppa is a PhD student and a cardiologist in IRCCS Azienda Ospedaliero-Universitaria di Bologna.
